# Diagnosis and treatment of tremor in psychiatric patients

**DOI:** 10.1192/j.eurpsy.2022.1841

**Published:** 2022-09-01

**Authors:** P. Van Harten

**Affiliations:** GGz Centraal, Scientific Research, Amersfoort, Netherlands

**Keywords:** Drug-induced, extrapyramidal, Tremor, Antipsychotics

## Abstract

**Introduction:**

Tremor is the most common movement disorder in adults. Due to the visibility, feelings of shame are often present. Many (psycho)pharmacological drugs can induce tremor or increase its severity as a side effect. Sometimes the burden of this side effect is greater than the burden of the psychiatric problem.

**Objectives:**

Knowledge of the different kinds of tremor in psychiatry, and the drugs that may be responsible. Differential diagnosis Treatment of tremor in psychiatry.

**Methods:**

A literature search on the most recent insights into classification, diagnosis, differentiation and treatment was carried out with emphasis on drug-induced tremor and its treatment.

**Results:**

The basic classification is resting, action and intention tremor. Tremors may be due to neurological and metabolic syndromes. Differentiation can often be made according to the time of onset, relation with starting or increasing the dosage of the medication and the course. Rest tremor is often related to antipsychotics and antiemetics and action tremor to lithium, antidepressants, valproic acid, and other anticonvulsants, but also to many drugs used in somatic conditions. The development of intention tremor should alarm the doctor because it could be an intoxication. Treatment of drug-induced tremor consists of reducing the dose or discontinuing the drug in question or switching to another drug with less risk of tremor. If this is not effective, adding a tremor suppressant may help (propranolol, primidone in action tremor and anticholinergics or amantadine in resting tremor.

**Conclusions:**

Tremor is a common side effect of many (psycho)pharmacological agents and treatment is often possible.

**Disclosure:**

No significant relationships.

